# Metabolic Regulation in the Development of Cardiovascular Disease and Heart Failure

**DOI:** 10.3390/ijms24108773

**Published:** 2023-05-15

**Authors:** Massimo Iacoviello

**Affiliations:** Department of Medical and Surgical Sciences, University of Foggia, Viale Luigi Pinto 1, 71100 Foggia, Italy; massimo.iacoviello@gmail.com

The Special Issue “Metabolic Regulation in the Development of Cardiovascular Disease and Heart Failure” focused on how metabolic diseases could cause a predisposition to cardiovascular diseases and, in particular, heart failure due to systolic or diastolic dysfunction or a combination thereof. Among metabolic disorders, impaired glucose metabolism is the metabolic condition most commonly associated with a greater incidence of both coronary artery disease and heart failure. However, there are also other very relevant aspects deserving of investigation related to the complex role of metabolic regulation in the different pre-clinical and clinical stages of heart failure. Moreover, the study of metabolic therapeutic approaches to treat patients with heart failure at different stages is a challenging field of research. Novel classes of hypoglycemic drugs, such as type 2 sodium-glucose cotransporter inhibitors (SGLT2i), have been shown to exert both cardio- and nephroprotection in patients with HF independently of the presence of diabetes.

In this complex scenario, the original article of Stratmann et al. [1] provides new information about the pathophysiological mechanisms by which hyperglycemia leads to HF in patients with diabetes. The authors evaluated the effects of long-term hyperglycemia on rat cardiomyoblasts overexpressing glucose transporter type 4 (GLUT4) (H9C2KE2). The authors used a proteomics-based analysis to evaluate glucose uptake, cell morphology changes, and apoptosis/necrosis measurements by flow cytometry. Moreover, they quantified brain natriuretic peptide (BNP) levels, the formation of reactive oxygen species (ROS), glucose consumption, and lactate production. After long-term exposure to hyperglycemia, H9C2KE2 showed an increase in GLUT4 presence on the cell surface that was associated with increased lactate production and an altered tricarboxylic acid (TCA) cycle with the accumulation of fumarate. Finally, an increase in BNP levels and oxidative stress, and lower antioxidant response, responsible for apoptosis and necrosis, were observed. All these results demonstrate that, in addition to the pathophysiological model related to insulin resistance, insulin-independent mechanisms related to hyperglycemia could lead to cardiometabolic abnormalities favoring the onset of diabetic cardiomyopathy. In fact, hyperglycemia and glucose overflow, such as that which occurs in the early phase of diabetes mellitus, could be responsible for metabolic stress affecting the TCA cycle at the fumarase level, causing a probable loss of NADH and inefficient energy production.

A complete picture of pathophysiological mechanisms in patients affected by type 2 diabetes mellitus is reported in detail in the review of Andreadi et al. [2]. The authors highlight the relevant role of inflammatory cytokines and oxidative stress, which, in the pre-diabetic phase, could favor the onset of type 2 diabetes mellitus. Moreover, they focus on the effects of insulin resistance and chronic hyperglycemia in determining micro- and macrovascular complications (cardiovascular disease, heart failure, hypertension, chronic kidney disease, and atherosclerosis). The review also discusses the effects of new therapeutic approaches, such as the use of dipeptydilpeptidase-IV inhibitors (DPP-IVi), which help to reduce the persistence of chronic hyperglycemia, glucagon-like protein receptor agonists (GLP1 RAs), which provide vascular protection, and the above mentioned SGLT2 inhibitors.

The beneficial effects of this class of drugs may be better understood by consulting the review of Gronda et al. [3], which highlights the pathophysiology of chronic kidney disease in patients with diabetes. Hyperglycemia and the related increased exposure of proximal tubular cells to filtered glucose are responsible for the overexpression of type 2 sodium-glucose cotransporter (SGLT2), which is responsible for the increased reabsorption of both glucose and Na+. The decreased amount of Na+ delivered to the juxtaglomerular apparatus (JGA) reduces local adenosine production and modulates the renin-angiotensin II axis. This leads to glomerular hyperfiltration related to the vasodilation of the afferent arteriole and increased tone of the efferent arteriole. Renal hyperfiltration increases renal O2 consumption, which is mainly related to Na+ and water reabsorption. In this setting, the SGLT2 inhibitors, by inhibiting both glucose and Na+ reabsorption, can increase the delivery of Na+ at the level of the JGA, thus inducing an increase in adenosine production, an increased tone of the afferent arteriole, a reduction of hyperfiltration and, consequently, of renal O2 demand and neuroendocrine activation. Interestingly, these mechanisms can explain the initial dip in the glomerular filtration rate (GFR) after the beginning of SGLT2i treatment, which is, in turn, followed by the stabilization of the GFR and a significant reduction in chronic kidney disease progression. Finally, there is a close correspondence between the dip in GFR after SGLT2i therapy and the significant reduction in heart failure events in randomized controlled trials of heart failure patients. Indeed, the initial dip in the GFR should be considered as the expression of SGLT2i therapy’s efficacy and its cardiac and renal protection.

If Gronda et al.’s review focuses on SGLT2 inhibitors, the review of Belli et al. [4] discusses the possible relevance of GLP-1 RAs. GLP-1 RAs have demonstrated the ability to reduce cardiovascular events (cardiovascular death or non-fatal myocardial infarction or stroke) in patients with diabetes beyond their hypoglycemic effects. At the vascular level, GLP-1 RAs can reduce oxidative stress, inflammation, and consequently, the formation and progression of atherosclerotic plaque. GLP-1 RAs can also exert other effects, such as a reduction in weight, adiposity, and blood pressure, which can not only further explain vascular protection, but can also represent mechanisms leading to an improvement in left ventricular diastolic function, as demonstrated by several studies. These effects could be useful in the treatment of patients who are obese with HF with preserved left ventricular ejection fraction (HFpEF), thus supporting further investigations aimed at assessing this hypothesis.

The review of Kruszewska et al. [5] further strengthens this hypothesis by describing the close relationship between obesity and cardiac remodeling and fibrosis, which favor the development of HFpEF. In this setting, in animal models with high-fat diet-induced obesity, several studies showed an increase in heart fibrosis due to collagen accumulation in the extracellular matrix (ECM). The consequent increase in myocardial stiffness is responsible for impaired diastolic function and the occurrence of HFpEF. The review accurately describes the complex processes of cardiac fibrosis, i.e., the ‘reparative’ process (which replaces necrotic heart tissue, such as after an ischemic injury) and the ‘reactive’ process (related to pathological hyperactivity of fibroblasts as a consequence of other conditions such as pressure overload, aging, or metabolic disturbances). The reactive process can be perivascular or interstitial, related to the accumulation of ECM proteins and significant cardiomyocyte loss. The different pathophysiological pathways that can mediate these changes in ECM are also discussed. In particular, the activation of the renin-angiotensin-aldosterone system and the sympathetic nervous system’s overactivity, systemic inflammation and oxidative stress, the increased clearance of natriuretic peptides, the reduced excretion of adiponectin and increased levels of leptin, and hyperinsulinemia could all be responsible for cardiac fibrosis in obese subjects. Finally, obesity and cardiac fibrosis could promote the onset of cardiovascular comorbidities such as atrial fibrillation, which further favors HFpEF.

The prediction of atrial fibrillation in patients receiving extracorporeal circulation after cardiac surgery is the subject of the original article of Altieri et al. [6]. Postoperative atrial fibrillation is very frequent for patients undergoing conventional extracorporeal circulation after surgery, but its pathophysiology needs to be better clarified. In their study, Altieri et al. demonstrated that among patients with atrial fibrillation, serum ferritin levels were significantly higher, as well as red cell distribution width and platelets. However, only ferritin remained associated with arrhythmia after correction for other confounding variables. The combination of the presence of high ferritin and P wave abnormalities or structural heart disease could represent a useful clinical approach in order to detect patients at higher risk of atrial fibrillation after cardiac surgery and to plan the most appropriate therapeutic strategy with anticoagulants.

The Special Issue also includes two interesting papers highlighting the possible role of new biomarkers. The original article of Szabó et al. [7] evaluated the role of pituitary adenylate cyclase-activating polypeptide (PACAP) and its specific PAC1 receptor in the plasma and myocardial tissue of patients with heart failure (HF). The biologically active forms of PACAP have anti-apoptotic, anti-ischemic, and anti-inflammatory effects. Moreover, they have an inhibitive effect on myocardial fibrosis, oxidative stress, and apoptosis. In order to further elucidate the relevance of PACAP, the authors measured the alterations of plasma PACAP-38-like immunoreactivity (PACAP-38 LI) in acute and chronic HF caused by ischemic or non-ischemic cardiomyopathy compared to age-matched healthy controls. The higher plasma levels of PACAP were observed in acute HF, but levels were lower in chronic HF. In patients with chronic HF, a significant negative correlation was observed between PACAP and NT-proBNP levels, whereas the correlation was positive in patients with acute HF. Interestingly, PACAP levels in myocardial tissues were lower in patients with end-stage HF. These results offer new information about the behavior of this biomarker in different types of HF, thus suggesting the need for further studies to better understand the usefulness of this biomarker in routine clinical practice.

The review of Sygitowicz and Sitkiewicz [8] focuses on the relevance of non-coding RNA fragments, specifically circular RNA (circRNA), in the development of HF. CircRNAs are differently expressed in the organs as well as in physiological and pathological conditions. They are involved in the silencing of transcription and translation and the suppression of definite mRNAs. Various studies have demonstrated that circRNAs play a relevant role in cardiomyocyte hypertrophy, fibrosis, autophagy, and apoptosis, thus favoring the development of HF. This evidence presents relevant implications because circRNA could be a promising biomarker. This possibility is also supported by their structural stability and their wide distribution both in cells and in the extracellular space. Moreover, circRNAs can act in an autocrine, paracrine, and even endocrine fashion, thus representing potentially useful targets in the treatment of cardiovascular diseases. Future studies should explore the potential diagnostic, prognostic, and therapeutic role of circRNAs.

Finally, the original article by Chang et al. [9] focuses on the role of blood reflux and metabolic regulation in the development of chronic venous disease. Histone deacetylases (HDACs) and DNA methyltransferases (DNMTs) are epigenetic factors involved in the metabolic signaling associated with endothelial dysfunction and atherosclerosis. The study demonstrates how HDACs and DNMTs are overexpressed in the endothelium of varicose veins with blood reflux and that this overexpression is even more evident in varicose veins with a longer duration of blood reflux. On the other hand, these epigenetic factors are under-expressed in the endothelium of veins with normal flow. The results of the study strengthen the possible role of HDACs and DNMTs in the genesis of cardiovascular diseases.
